# Topological data analysis expands the genotype to phenotype map for 3D maize root system architecture

**DOI:** 10.3389/fpls.2023.1260005

**Published:** 2024-01-15

**Authors:** Mao Li, Zhengbin Liu, Ni Jiang, Benjamin Laws, Christine Tiskevich, Stephen P. Moose, Christopher N. Topp

**Affiliations:** ^1^ Donald Danforth Plant Science Center, St. Louis, MO, United States; ^2^ Department of Crop Sciences, University of Illinois at Urbana-Champaign, Urbana, IL, United States

**Keywords:** topological data analysis, persistent homology, multivariate analysis, 3D root system architecture, GWAS, genotype to phenotype, phenome

## Abstract

A central goal of biology is to understand how genetic variation produces phenotypic variation, which has been described as a genotype to phenotype (G to P) map. The plant form is continuously shaped by intrinsic developmental and extrinsic environmental inputs, and therefore plant phenomes are highly multivariate and require comprehensive approaches to fully quantify. Yet a common assumption in plant phenotyping efforts is that a few pre-selected measurements can adequately describe the relevant phenome space. Our poor understanding of the genetic basis of root system architecture is at least partially a result of this incongruence. Root systems are complex 3D structures that are most often studied as 2D representations measured with relatively simple univariate traits. In prior work, we showed that persistent homology, a topological data analysis method that does not pre-suppose the salient features of the data, could expand the phenotypic trait space and identify new G to P relations from a commonly used 2D root phenotyping platform. Here we extend the work to entire 3D root system architectures of maize seedlings from a mapping population that was designed to understand the genetic basis of maize-nitrogen relations. Using a panel of 84 univariate traits, persistent homology methods developed for 3D branching, and multivariate vectors of the collective trait space, we found that each method captures distinct information about root system variation as evidenced by the majority of non-overlapping QTL, and hence that root phenotypic trait space is not easily exhausted. The work offers a data-driven method for assessing 3D root structure and highlights the importance of non-canonical phenotypes for more accurate representations of the G to P map.

## Introduction

The importance of root system architecture (RSA) to plant health and productivity has been well established. Rational design of root traits that are tailored to the environment is an appealing approach to generating more efficient, productive plants with less negative environmental impact (White et al., 2013). Yet little is known about the quantitative genetic control of root system architecture, particularly outside of model systems and laboratory environments. The most advanced work is from rice, where two seminal genes were identified from field studies: Deeper rooting 1 (Dro1) conferring drought avoidance (Uga et al., 2013), and Phosphorus-starvation tolerance 1 (Pstol1) controlling a low phosphorus tolerance QTL (Gamuyao et al., 2012). In maize, a major target is to increase the efficiency of applied nitrogen fertilizer capture, but despite strong associations with root system architecture QTL (Li et al., 2015), only recently have underlying genes been identified that may provide durable nitrogen uptake benefits in agricultural systems (Schneider et al., 2021).

The Illinois Long Term Selection Experiment for Protein began in 1896 as a recurrent selection scheme for increased or decreased maize grain protein concentration (Moose et al., 2004). As grain protein derives from plant accumulated nitrogen, selection for grain protein concentration has also impacted other phenotypes associated with nitrogen uptake and partitioning (Uribelarrea et al., 2007; Uribelarrea et al., 2009). A population of recombinant inbred lines (the IPSRIs) derived from the divergently selected Illinois High Protein (IHP) and Illinois Low Protein (ILP) strains has previously been described that varies significantly for grain protein concentration (Lucas et al., 2013). We hypothesized that if root system architecture is important for nitrogen uptake in the field, root traits would have been indirectly selected on, and mapping the genes involved could lead to a better understanding of efficient maize N-uptake.

The central goal of phenomics is to explain more of the observed phenotypic variance by genetic factors and thereby expand the genotype-to-phenotype map (Houle et al., 2010). A primary limitation to this work is the low information content of many phenotyping approaches, including the use of serial predefined univariate traits to describe complex phenotypes (Pitchers et al., 2019). We previously conducted a quantitative genetic mapping analysis of 3D root system architecture using a similar optical gel-based growth and imaging system as was used in this study (Topp et al., 2013). A key finding was that using multivariate composite traits derived from constituent univariate traits could identify regions of the rice genome with large effects on RSA that were not identified by the univariate traits alone. Yet this study still relied on predefined traits to measure a complex phenotype (RSA) that we have little scientific understanding of. Subsequently, we showed how data-driven traits could enhance traditional univariate features in measuring leaf and 2D root traits for enhanced G to P mapping (Li et al., 2018a).

Persistent homology (PH) is a topological data analysis (TDA) method to infer complex data structure. Its mathematical theory can be traced back to the 1940s or even further (Morse, 1940). Since the early 2000s, PH has been efficiently computed to quantify topological features (Edelsbrunner et al., 2002), then TDA started to be broadly applied to many fields such as atomic structures, material science, cancers, sensors networks, among others (De Silva and Ghrist, 2007; Edelsbrunner and Morozov, 2013; Kramar et al., 2013; Chazal and Michel, 2021). But at that time, it had not been widely introduced in the analysis of plant phenotyping. In the works (Li et al., 2017; Li et al., 2018a; Li et al., 2019), we developed PH-based methods tailored to quantify complex 2D shapes and 3D branching architectures and applied them in plant phenotyping. Meanwhile, many other TDA methods have also been employed to this field such as Persistence Intensity Array (Medina and Doerge, 2016), Euler Characteristic Transform (Amezquita et al, 2021), mapper (Amézquita et al., 2020) and many others. Our previous study showed that PH can be used to capture comprehensive and complementary morphological features in 2D such as leaf shape and 2D root projections in tomato as evidenced by detecting more and unique QTL that provided a more complete understanding of genetic architecture (Li et al., 2018a). PH has also been applied to quantify 3D branched systems in an integrated manner that adds to the description and statistical discrimination of complex architectures (Li et al., 2017; Delory et al., 2018; Li et al., 2019). In this study, we built and expanded upon these methods for quantifying 3D root architecture. These works support a theory that expanding the phenotypic space can result in a fuller description of the actual plant phenome, which enhances our ability to map relationships to the underlying genotypic diversity.

Here we apply the methods to a quantitative genetic analysis of maize 3D RSA in the IPSRI mapping population using, initially, 84 univariate traits, PH methods developed for 3D branching, and multivariate vectors of those traits. We used a variance inflation factor (VIF) to reduce the collinearity of traits, and multivariate models to concentrate the variation along fewer dimensions. We expanded our previous PH methods with two mathematical functions to capture comprehensive summaries of complex root systems. The genetic architectures of these 3D root traits were queried using Genome Wide Association (GWA) mapping, and we found that PH traits identified loci that were otherwise undetected by the univariate and multivariate trait. Loci were analyzed to understand genetic variation for root system architecture that occurred indirectly as the result of selection for increased seed protein content.

## Result

### Workflow

**Figure 1:**
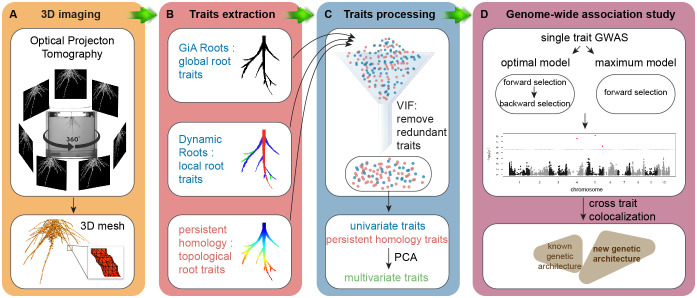
Workflow. **(A)** 3D imaging, optical imaging platform is used to get 3D root system grown in the gel; **(B)** trait extraction, global root traits are extracted by GiA Roots, local roots traits are measured by Dynamic Roots, and topological structure is quantified by a topological data analysis approach persistent homology implemented in MATLAB (R2017a); **(C)** trait processing, variance inflation factor (VIF, vif() in R) is used to remove the redundant traits. Then we perform principal component analysis (PCA, prcomp() in R) on the remaining traits to get PCs as multivariate traits; **(D)** genome-wide association study (GWAS), both optimal model and maximum model (MLMM in R, Segura et al., 2012) to detect the traits associated quantitative traits loci (QTL).

A non-destructive gel-based optical tomography imaging platform captured 3D RSA at day 9 after germination (**Figure 1A**). We used the RSA-GiA3D software to measure univariate root traits such as surface area, total root length and solidity (Galkovskyi et al., 2012; Topp et al., 2013), Dynamic Roots to measure local individual traits such as first order lateral root number and lateral root soil angle (Symonova et al., 2015; Jiang et al., 2019), and a TDA approach with PH to quantify the topology of the root structure (Li et al., 2017; Li et al., 2019; **Figure 1B**). Because some traits are mathematically and/or phenotypically correlated, we employed the statistical approach variance inflation factor (VIF, Stine, 1995) to remove the redundant traits. We chose VIF because it has been effectively used in high-throughput plant phenotyping studies for this purpose for at least a decade (Chen et al., 2014; Falk et al., 2020), although advances in the statistics of high-dimensional data analysis have since proposed more sophisticated methods that can increase understanding of the most important response variables (Cheng et al., 2022) (see Conclusion). The remaining traits after VIF are highlighted in **Supplementary Table 1** from the full list of all phenotypic traits. Then we performed principal component analysis (PCA) on the remaining univariate traits and PH traits and treated PCs as the multivariate traits (**Figure 1C**). Like our rationale for using VIF, we chose to use PCA because of its common use in plant phenotyping studies (Duc et al., 2023), but subsequent variations such as sparse PCA may increase interpretability of the multivariate traits (Zou et al., 2006, see Conclusion). We made GWA using univariate traits, PH traits, and multivariate traits by a Multi-Locus Mixed Model (MLMM), evaluating both the optimal and maximum models (**Figure 1D**; Ziegler et al., 2018). We then compared the results by co-aligning across the maize genome and evaluating correspondences in different window sizes.

### A topological data analysis method: persistent homology

**Figure 2:**
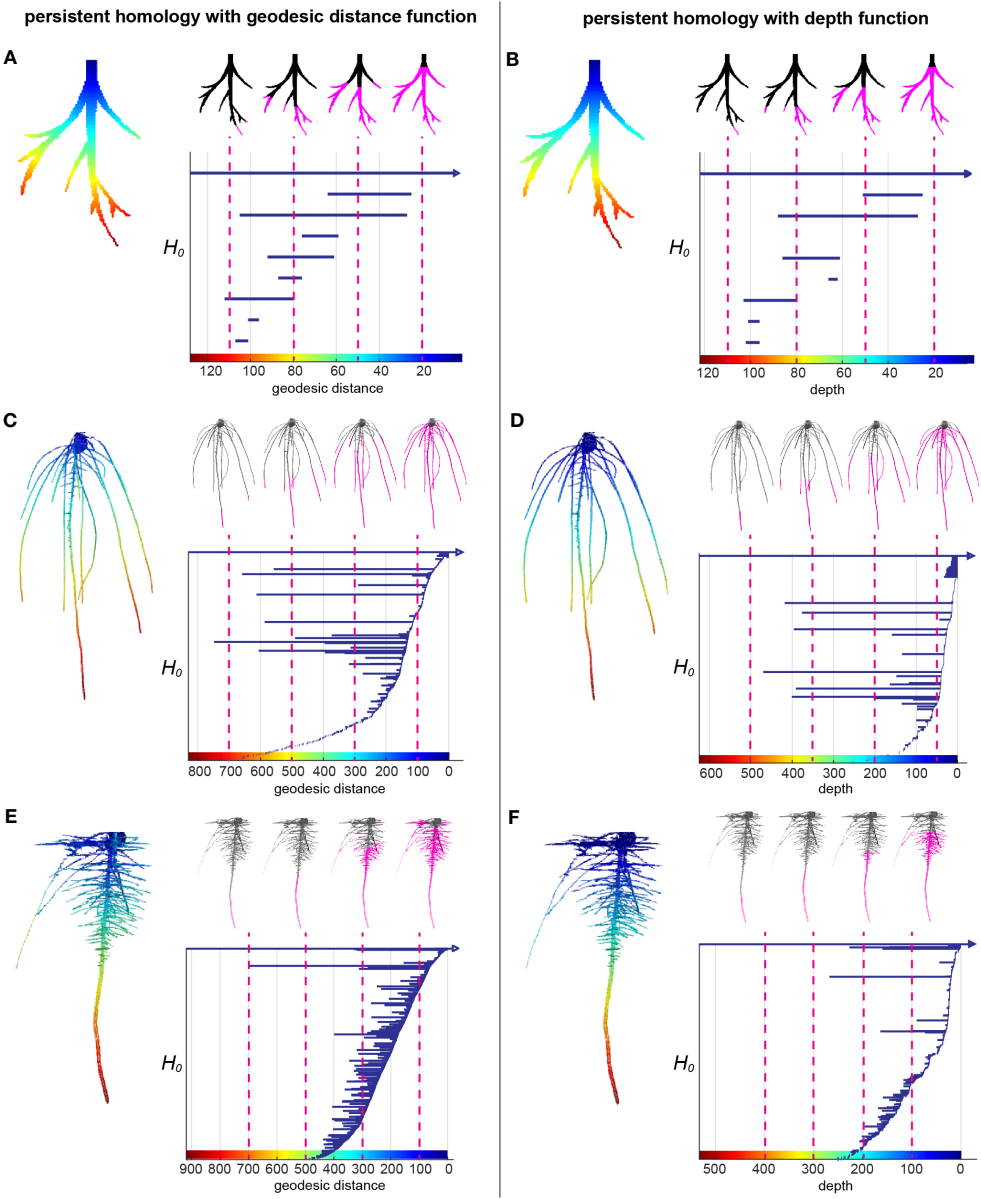
A topological data analysis method persistent homology to comprehensively quantify branching structure. **(A)** Heatmap of the geodesic distance function to the top assigned on each pixel of a simple root example. The greatest distance to the smallest distance are colored from red to blue (left panel). H_0_ persistent barcode records the “birth” and “death” of each connected component as the level set (the pixels that have greater distance than given a geodesic distance level at x axis) expanded from maximum distance to the minimum distance (right panel). **(B)** is similar with **(A)** but with depth function (straight distance from each pixel to the top) as the function. **(C, E)** are similar with **(A)** but with two root examples from this data showing different structures. **(D, F)** are similar with **(B)** but with two root examples from this data showing different structures.

Root systems are commonly measured by some intuitive topological descriptors such as number of tips and geometric descriptors such as root lengths and root-soil angles, which are useful but do not capture the entirety of 3D topological structure. Recently, TDA has demonstrated its wide application and success to extract complementary and comprehensive plant phenotypic traits such as 2D leaf shape, 2D root architecture and 3D inflorescence architecture (Li et al., 2017; Li et al., 2018a; Li et al., 2019). We used the TDA method PH to quantify the 3D RSA more comprehensively. We first extracted the surface voxels from the 3D model and manually cleaned the topological noise (e.g. branching touches) as much as we could. Then we assigned each surface voxel a value showing the curved distance from the voxel along the root to its top at the surface of the gel, which is called geodesic distance. Geodesic distance provides us the near true length of each root which is biologically important and known to be useful to distinguish different root architectures. The root examples in the **Figures 2A, C, E** show the colormap of the geodesic distance where red indicates high value, blue means low value. Starting from the voxel with the largest geodesic distance value, we plotted a bar to record the connected component information. Every isolated piece/blob is treated as one connected component. Then we kept adding new voxels as we continuously decrease the geodesic distance level. If the added voxels are connected to one of the components, the bar corresponding to that component will elongate; if the added voxels are the beginning of a new component (tip of a root), we start to plot to a new bar with the birth at the corresponding geodesic distance level; and if the added voxels merge two components, the shorter bar will die and the longer bar will elongate. At the end, the distance level is decreased to 0 and all the bars die except one since the root system is a single connected component. The bar graph forms a persistence barcode to record the topological information of the root structure (**Figures 2A, C, E**).

The persistence barcode associated with geodesic distance can record the topological structure and also some geometric information. For example, the number of bars is equal to the number of roots. The birth and death of each bar shows the depth of the root tip and emergence. The length of each bar indicates the length of the branch. However, it does not measure the angle. Thus, we combined it with a second persistence barcode that included a depth function to capture some of the angle information (**Figures 2B, D, F**). Because the depth of the root tip is highly related to the angle of the root relative to the soil surface, horizontal roots will have much shorter bars in the depth barcode (**Figure 2F**). For convenience, we simply name the two different persistence barcodes as geo-barcode and depth-barcode.

Barcodes need to be quantitatively compared to measure the similarity, quantify the variation and other statistics. Bottleneck distance is a robust metric to measure the distance between any two persistence barcodes (Cohen-Steiner et al., 2007). Intuitively, it measures how much minimum energy that it needs to take for reassembling a barcode to be the same as the other barcode. For each root, we computed the geo-barcode and depth-barcode. We measured the similarity between two roots by the square root of summation of bottleneck distance squared between two geo-barcodes and bottleneck distance squared between two depth-barcodes:


$$\sqrt{d^{2}\left(geobarcode_{1},geobarcode_{2}\right)+d^{2}(depthbarcode_{1},depthbarcode_{2}})$$

This measurement itself is a distance due to Cauchy-Schwarz inequality. Then we calculated the similarity matrix for the entire population and performed a multidimensional scaling method to get MDS scores using MATLAB function *cmdscale().* For a non-Euclidean distance matrix, MDS will map the data into Euclidean space, while preserving the pairwise distance as well as possible. Then we performed PCA on the MDS scores which returned the same scores, but also provided some additional information such as the percentage variance per PCs. Thus we named it as PHGeodesicDepth_MDSPCs (GH_PCs). These GH_PCs are treated as PH traits. Similarly, G_PCs are the PH traits when only using geo-barcode to compute the similarity matrix. Please see step 5 of persistent homology in the Method section.

We introduced persistence barcode because it is more intuitive for biologists to understand. But the following feature is more straightforward to be calculated from another descriptor, persistence diagram. Persistence diagram is an equivalent descriptor to the persistence barcode (**Supplementary Figure 1A, B**). It is a 2D scatter plot: along the x-axis is the birth value for each bar and along the y-axis is the death value, unioning with the diagonal line. We turned this scatter plot into a Gaussian density estimator (**Supplementary Figure 1C**, Adams et al., 2017) and performed PCA on the vectorized Gaussian density estimator. The PC scores are treated as another set of PH traits, named as PHDiagramKDE_PCs (PDD_PCs). **Table 1** lists all the PH traits and their descriptions. In total, we have three groups of PH traits: G_PCs, GH_PCs, and PDD_PCs. G_PCs are the MDS-PC scores derived from the bottleneck distance of geo-barcode, GH_PCs are the MDS-PC scores derived from the combined distance of both geo-barcode and depth-barcode, PDD_PCs are the PC scores of the vectorized density estimator from geodesic persistence diagram.

**Table 1:** Persistent homology traits.

PH traits	Abb.	Description
PHGeodesicDepth_MDSPCs	GH_PCs	MDS-PCA for persistent homology with the combined geodesic distance function and depth function
PHGeodesic_MDSPCs	G_PCs	MDS-PCA for persistent homology with geodesic distance function
PHDiagramKDE_PCs	PDD_PCs	PCA for Gaussian density estimator of persistence diagram with geodesic distance function

### Trait processing and visualization

**Figure 3:**
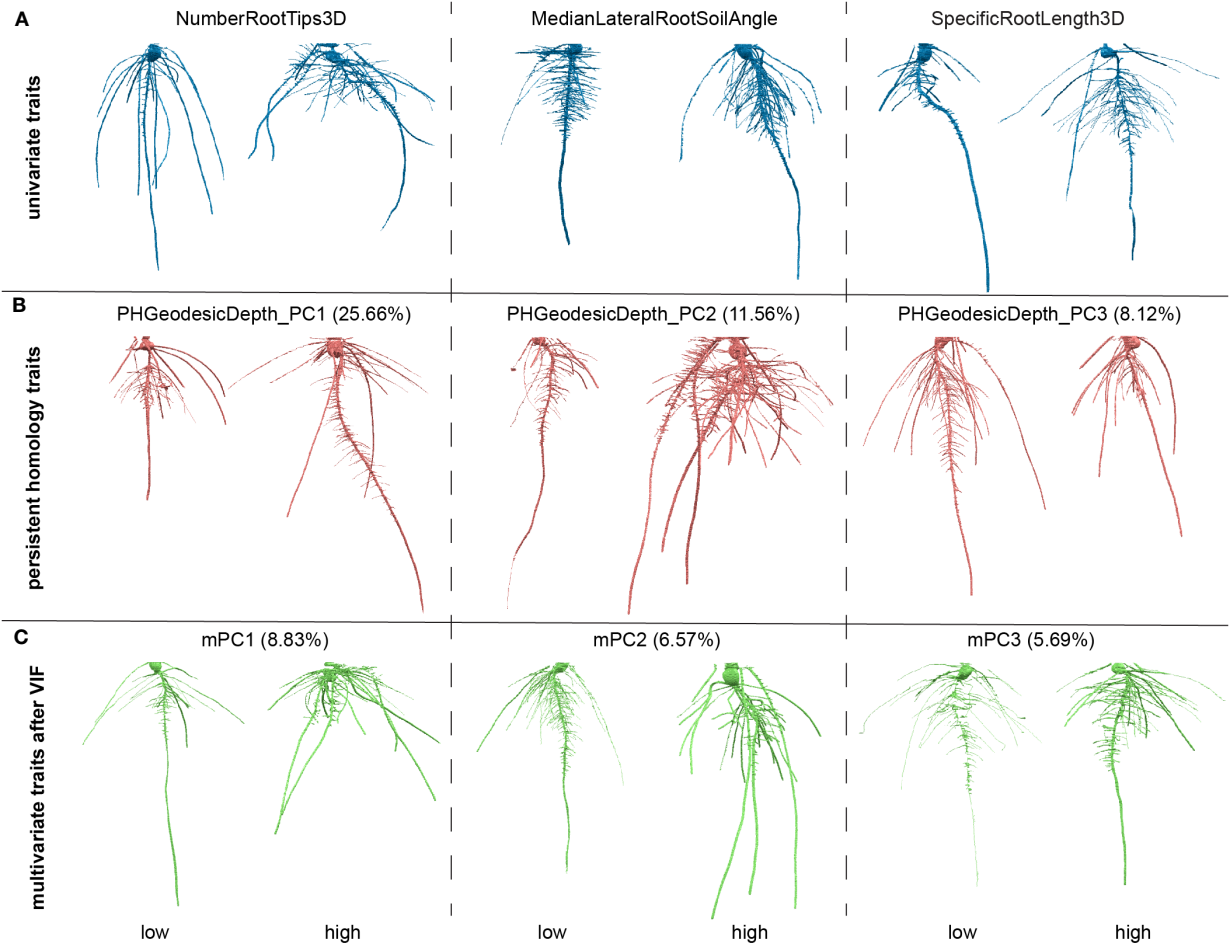
Examples of roots for high and low values for traits. **(A)** Examples for three of the univariate traits, number of root tips, median angle between the lateral root and soil, specific root length; **(B)** examples three of the persistent homology traits, first three principal components (PCs) for persistent homology with geodesic distance and depth functions; The percentage is the variance explained from that PC in persistent homology traits. **(C)** Examples for three of the multivariate traits, first three PCs for the traits after variance inflation factor (VIF). The percentage is the variance explained from that PC in all measured features.

Some traits could be strongly correlated. To reduce collinearity among explanatory variables (**Supplementary Figure 2A**), we applied a VIF. Simply, VIF_j_ = 1/(1-R_j_^2^) where the VIF for variable j is the reciprocal of the inverse of R^2^ from the regression. To pick a proper threshold (see Materials and Methods), we calculated a sequence of thresholds and recorded the number of remaining traits and treated the thresholds as x values and remaining trait numbers as y values. We observed this data can fit well with a logarithm function (y=a*ln(x-b)+c, where a=15.71, b=0.8853, c=57.88, **Supplementary Figure 1B**). We found the threshold at slope = 1 which means the VIF threshold increases at the same rate with the increasing of the traits number. We treated it as a high threshold. We picked a median threshold which is half of this threshold (value = 8; **Supplementary Figures 2B, C**). Note that the first 29 G_PCs were removed because they are highly correlated to GH_PCs. The VIF chose to keep GH_PCs rather than G_PCs which may imply the depth function contributed to capture features (e.g. angle difference) that makes GH_PCs to be less correlated to other features. G_PC30 to G_PC36 were kept which may imply the depth function and geodesic distance captured some detailed differences which have much smaller variance compared to major topological differences. Then we performed PCA on the remaining traits including commonly used geometric univariate traits and PH traits. The output PC scores are recorded as mPCs, multivariate traits. The PH traits as well as multivariate traits are comprehensive but less intuitive compared to commonly used univariate traits. One way to interpret what a trait measures is to look at the genotypes with extreme values for that trait, but where other traits that are close to their mean values (**Figure 3**). We also plotted out the distribution for each of the VIF remaining traits and first five mPCs to show the normed spread of the data (**Supplementary Figure 3**).

### Expanding the genotype to phenotype map for maize root system architecture

Previous studies reported that the Illinois High Protein inbred (IHP1; derived from the IHP cycle 90 population) exhibits elevated N uptake and assimilation relative to the Illinois Low Protein inbred (ILP1; derived from the ILP cycle 90 population) (Moose et al., 2004; Lucas, 2013). By observing the RSA of both lines in gel, we found that IHP1 seedlings have nearly twice as many lateral roots than ILP1 with a high statistical significance (p=0.0002; **Supplementary Figure 4**). This finding is congruent with root adaptive responses to nitrogen availability (Drew et al., 1973; Robinson et al., 1999) and suggests that RSA may indeed have been inadvertently changed during recurrent selection for seed protein.

**Figure 4:**
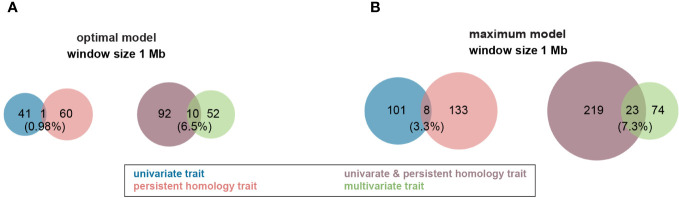
Venn diagram of profile of trait-associated SNPs (TAS) with 1 Mb window size across all traits in both Multi-Locus Mixed Model (MLMM). **(A)** TASs were identified with optimal model among all different traits. **(B)** More TASs were identified with maximum model among all different traits. Groups of traits were color coded in the figure. .

**Figure 5:**
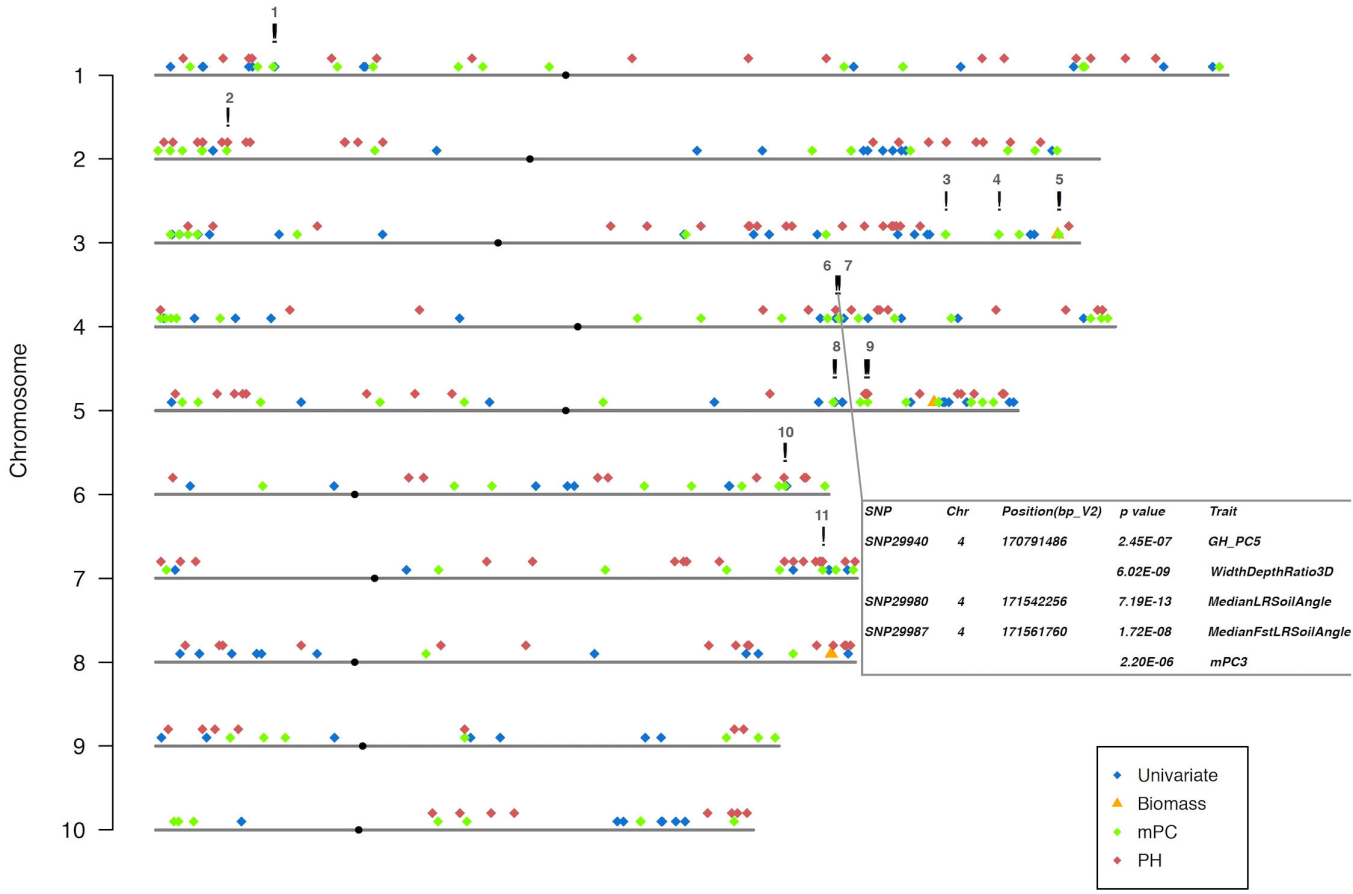
The genetic architecture of all the measurements in the study. TASs were identified from MLMM optimal model with 1.0 x e-5 as the threshold. Table in the bottom right highlighted the two clusters of co-localized TASs (!) on maize chromosome 4. SNP names, their physical positions on maize genome, their p-values and corresponding traits were illustrated in the table. All the TASs are color coded based on its corresponding trait groups. Unlike others, biomass TAS is also represented with a different symbol (▵). Centromeres on each chromosome are labeled (**·**) and their positions referred to the publication (Schneider et al., 2016).

To further explore this possibility and map the genetic basis of root phenotypes using our expanded phenome, we performed a Genome Wide Association Study (GWAS). GBS (genotyping by sequencing; Elshire et al., 2011) was used to generate, 60,418 SNPs (single nucleotide polymorphisms). Using MLMM (Multi-Locus Mixed Model; Segura et al., 2012), one stringent model was chosen as the final/optimal with two different ways for multiple comparison, Bonferroni correction and E-BIC (Segura et al., 2012). The optimal model was picked after evaluating a few models with both forward and backward stepwise regressions. However, a few studies have shown that the Bonferroni correction increases the probability of producing false negatives (Segura et al., 2012; Ziegler et al., 2018). Therefore, we also employed a modified maximum model with only the forward stepwise regression and present both results (**Figures 4**, **5**; **Supplementary Figure 5**, **Supplementary Tables 2**, **3**).

For the optimal model, using a 1Mb window size (Yang et al., 2015b; Hu et al., 2017) as the QTL co-localization boundary, 102 total loci were detected for PH and all univariate traits in this study. Among them, only one 1Mb chromosome region is shared by both, representing <1% of QTL (**Figure 4A**). This locus (SNP29940) is located at ~170Mb on maize chromosome 4 (B73_Ref_v2) corresponding to PH traits, GH_PC5 and WidthDepthRatio3D (**Figure 5**). The SNP hits the second exon of the ethylene receptor ETR2-like gene (GRMZM2G075368), also known as the *ZmETR3*, which was previously shown to be involved in root growth by regulation of ABA and/or auxin accumulation in root tips (Yang et al., 2015a; Li et al., 2018b). In addition, the same region was also found involved in regulation of another univariate median lateral root soil angle and the multivariate mPC3. although not the same SNP (**Figure 5**, SN29980, SNP29987). As described above, PCA was performed to generate multivariate traits (**Supplementary Figure 2**). 62 QTL were identified for those multivariate traits with optimal model in MLMM (**Figure 4A**). 10 of these co-localized with the 102 loci identified in with the PH and univariate traits in the 1Mb window, representing 6.5% of total QTL. The maximum model identified more than twice as many QTL as did the optimal model, but only slightly increased the percentage of QTL shared by univariate and PH (0.98% to 3.3%) or multivariate and univariate + PH (6.5% to 7.3%; **Figure 4B**). The list of trait-associated SNPs (TAS) for each phenotype class, model, and 1Mb overlaps thereof are provided in **Supplementary Tables 2**–**4**. We also performed a similar analysis with no window size and got the majority of non-overlapped QTL (**Supplementary Figure 5**). These results provide evidence of an expanded G to P map using both a TDA and a multivariate statistical approach, which are complementary and not exclusive.

**Figure 6:**
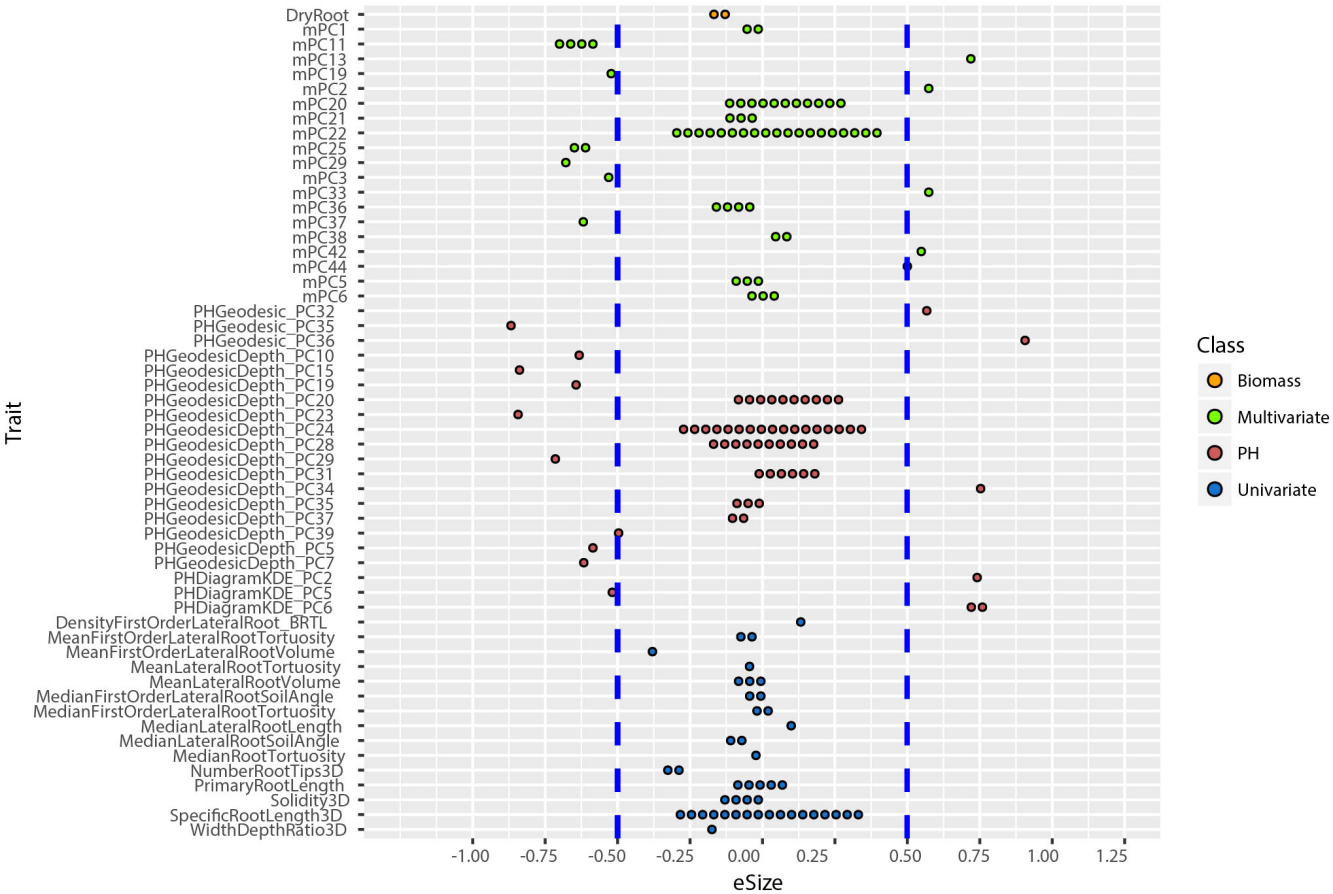
Distribution of allele effect size of all the TASs identified in MLMM optimal model. Traits are labeled as y-axis and x-axis indicates their corresponding allele effect size (eSize). Different color codes represent different groups of traits in the study.

An allelic effect size is how much of the total variation for a given phenotype the allele explains in the statistical model, and its direction (positive is larger and negative is smaller numerical value). Prior work showed that including multivariate and TDA of phenotypes could not only identify new loci (**Figure 4**), but also loci of large effect size (Topp et al., 2013; Li et al., 2018a). To compute the effect size and direction in for each trait, we averaged the major allele trait values first, then we divided the estimate values of each QTL by the average values [effect size =estimate/mean(major allele)]. In this way, the positive value indicates increases on the major allele while negative values indicate increases on the minor allele phenotype. We found that most univariate traits have a narrow range of allele effect size (-0.25 to +0.25) while the effect size of both PH and multivariate traits ranged widely (-0.91 to +0.90) (**Figure 6**; **Supplementary Figure 6**). These results reinforce the enhanced ability of data-driven phenotypes to identify large-effect QTL.

## Conclusion

Root systems are the primary interface of plants with the soil and are foundational for the basic physiology of water and nutrient capture as well as for shaping the rhizosphere and biogeochemical processes therein. Most modern crop varieties have been domesticated and bred without root system function or efficiency in mind, including with what can now be viewed as unsustainable water and fertilizer inputs. Yet, with a few notable exceptions (Gamuyao et al., 2012; Uga et al., 2013; Schneider et al., 2021; Ren et al., 2022) root biologists and plant breeders have a poor understanding of genes that control quantitative root system architecture traits, and therefore lack means to rationally design and test proposed root system ideotypes (Lynch, 2013).

The field of phenomics is tasked with building the genotype to phenotype map, and a wide array of root phenotyping technologies have been reported recently, most using various forms of imaging (Atkinson et al., 2019). But while our abilities to capture relevant root phenotype information at scale have increased, the analytical approaches have not grown to a similar extent, with most of the phenotypic space explored by a set of human-intuited reductionist metrics that may miss emergent properties (Li et al., 2018a) and other cryptic features (Chitwood and Topp, 2015) of the data. In this work we revealed that a genetic architecture of 3D maize root structure can be enhanced by a more data-driven approach. We conducted GWA comparing the SNPs identified from a comprehensive suite of univariate computer vision traits, a new mathematical method, persistent homology (PH) – a topological data analysis method that does not pre-suppose specific important features, as well as multivariate vectors of those traits. We show largely separate genetic architectures using these methods, suggesting that the complexity of maize 3D root system architecture phenotypes is not adequately captured by current commonly used metrics. Furthermore, PH features can have much larger effect sizes than univariate and derived multivariate metrics, suggesting the specific underlying genetic variation could be more easily identified and tested for function toward ideotype development. Thus, topological data analysis expands the genotype to phenotype map in the 3D maize root system.

The functions (geodesic and depth functions) used for persistent homology are length based. We used the same unit for all our samples which means the persistence diagrams are comparable. Persistence diagrams are not robust to scaling or metrics, if other research groups have the data with the same scale and metrics of their samples, our methods and results can be used to compare with their findings. On the other hand, the results of dimension reduction methods such as VIF and PCA that are commonly used in plant phenotyping research can be less explicitly comparable across studies. For VIF, the selected response variable can be somewhat labile for highly colinear traits such that different analyses produce similar but not identical results (for example, selecting surface area versus volume, or vice-versa). The Mutual Information-Variance Inflation Factor (MI-VIF) has been proposed to improve variable selection, by additionally maximizing the correlation between the independent and response variables using MI theory when testing for multicollinearity using VIF (Cheng et al., 2022). The results appear promising for spectral data, but to what extent MI-VIF may provide more robust selection of shape variables in plant phenotyping research is an interesting future research question. Likewise, the loadings of standard principal components derived from high-dimensional and partially multicollinear datasets can also be labile and dense (many features contribute small amounts to many PCs), making interpretability of the underlying variation a challenge. Sparse Principal Component Analysis (SPCA) is a family of methods that seek to sparsify the number of features in each PC through penalizations or constraints, for example lasso penalized least-squares (Zou et al., 2006; Guerra-Urzola et al., 2021). Given the number of possible approaches to sparsifying PCA and the fact that they can lead to non-equivalent solutions, widespread application of SPCA to high-throughput plant phenotyping will likely require substantial development. These considerations, along with the results presented here, suggest the field of plant phenomics has much additional work to do both empirically and theoretically to fully realize the genotype to phenotype map.

## Materials and methods

### Plant material

The maize IPSRI (Illinois Protein Strain Recombinant Inbreds) mapping population was initially described in Lucas et al. (2013). Initially, five plants from cycle 70 of Illinois Low Protein were crossed to five plants from cycle 70 of Illinois High Protein. After seven generations of random mating 200 individuals to reduce linkage disequilibrium (Dudley et al., 2007), 500 randomly selected individuals were self-pollinated for six generations. The 500 resulting recombinant inbred lines were genotyped with a set of 500 SNPs as described in Dudley et al. (2007). Clustering analysis using Euclidean distance of variation in marker genotypes produced 138 distinct groups where one IPSRI line was selected from each group to form a representative core set for phenotyping. These 138 core IPSRI inbreds were used in the root phenotyping analysis presented here. On average each genotype was replicated 3 times (**Supplementary Figure 7**).

Seed preparation and growth conditions followed Jiang et al. (2019): “the growth medium was made with a modified 1/2x Hoagland solution pH 6.0 and solidified with gellan gum. The seeds were sterilized with 35% hydrogen peroxide for 20 minutes and rinsed four times with RO (reverse osmosis) water. After imbibing in RO water for 8 hours at 29°C in the dark, the seeds were sterilized again with 35% hydrogen peroxide for 10 minutes and rinsed four times with sterile water. The seeds were germinated at 29°C in the dark until the radicle reached 1-2 cm in length, approximately 48 hours. One seedling was planted into a glass growth cylinder sealed with Saran wrap - this constitutes a biological replicate. The cylinders were placed on a dark shelf at ambient conditions overnight for acclimation before moving them into a growth chamber starting at 4 DAG. The plants were lit with 315W Philips Ceramic Metal Halide bulbs, with a light intensity at the top of each jar of 700 µmol/m²/s. Humidity in the chamber was maintained at 50%, although the jars were sealed with Saran wrap. Temperatures were set to 28°C during the day and 24°C at night, with a 16/8h day/night cycle”.

### Imaging platform and software

As described in Griffiths et al. (2023), but with a different lens: the imaging setup consisted of an Allied Vision Manta G-609 machine vision camera (Allied Vision Technologies GmbH, Stadtroda, Germany) with a Kowa LM50SC 50mm 1” f/2.0 lens (Kowa, Japan) and an electronic turntable. The turntable operated in a water-filled tank to correct for light diffraction when imaging the glass cylinders. The glass cylinders were partly submerged to when placed in the center of the turntable. An LED flat panel light was used as a backlight to produce near binary images of the roots with a black silhouette of roots in the foreground against a white background. Root imaging took approximately 2 min per plant with 72 images collected over a 360-degree rotation. We studied 520 3D models and used the RSA-GiA3D software to measure global root traits (Galkovskyi et al., 2012; Topp et al., 2013) and the Dynamic Roots software to measure local individual traits (Symonova et al., 2015; Jiang et al., 2019). For the TDA analysis, we first extracted the surface voxel from the 3D model and saved them as.ply files. Then we manually cleaned some of the topological noises such as branching touches and loops as much as possible in Meshlab (Cignoni et al., 2008).

### Persistent homology

The pipeline for converting 3D root imaging data into persistent homology traits could involve five main steps: Extracting network (e.g. vertices and edges connecting vertices) from images, Assigning function values onto the network, Computing persistent barcodes with a filtration, Computing pairwise bottleneck distance for entire population, Performing statistical approach such as multidimensional scaling. The pipeline was conducted in MATLAB R2017a (The MathWorks Inc, 2017). A similar approach and code can also be found in Li et al. (2019).

1. Extracting network from images. More strictly speaking, we need to form a simplicial complex (a mathematical term which consists of vertices, edges, triangle faces, tetrahedron and even higher dimensional “triangle” under some glue criterion). As the root should never have the loops, we only need to calculate H0-persistence barcode (i.e. connected component). We can just extract a network which only has vertices and edges connecting those vertices. Our 3D images are binary and we treat each root pixel as a vertex connecting it to its neighbor (if a pixel falls within its 3x3x3 cubes) by an edge.
2. Assigning function values onto the network. How to define a mathematical function is flexible. For the root data, we assigned each vertex a value showing the shortest distance in the network from this vertex to the top of the root. This is the geodesic distance which is the curved distance from that pixel along the root to the top. In our study, we also use another mathematical function, depth function, to incorporate more information such as angle. The depth function allows to assign each vertex the value showing the straight height to the top plane. After assigning values on the vertices, we assigned each edge a value which is the minimum value between the two vertices that this edge connects.
3. Computing persistence barcodes with a filtration. A filtration is a nested sequence of subnetworks which the later subnetwork always includes the former network. For example, for geodesic distance function, the start subnetwork is formed from the vertices and edges which have the maximum values (see the pink part of the root in **Figure 2A**). Then the next subnetworks is formed by decreasing a little threshold and adding the new vertices and edges which have equal or larger values than this threshold. In this analysis, we chose the minimal integer step size which is 1 voxel size. The H0-persistence barcode consists of bars showing the persistence of each connected component. Each bar has its birth value and death value. The birth value is the threshold where a new connected component appears in the subnetwork. The death value is the threshold where this connected component gets merged into another subnetwork. Each root system has one persistence barcode with geodesic distance function and one persistence barcode with depth function.
4. Computing pairwise bottleneck distance. Given any two persistence barcodes with geodesic distance function, the bottleneck distance can robustly measure the similarity between these two barcodes. It intuitively measures the minimum cost to move the bars in one barcode to resemble the other one. The pairwise bottleneck distance matrix for persistent homology with geodesic distance function can be computed for the population. Similarly, we also can compute the pairwise bottleneck distance matrix for persistent homology with depth function. Note that bottleneck distance can also be calculated by an equivalent descriptor, persistence diagram (**Supplementary Figure 1**). Persistence diagram is a 2D scatter plot: along the x-axis is the birth value for each bar and along the y-axis is the death value, unioning with the diagonal line. In this analysis, we used bottleneck distance with a geodesic distance function first. Then we were motivated by its limitation (e.g. miss angle information) and expanded the method by combining it with depth function.
5. Performing statistical approaches. PCA cannot be directly performed on a non-Euclidean distance matrix, therefore given a Bottleneck distance matrix, we first perform multidimensional scaling (MDS) and then perform PCA on MDS scores to have both MDS (PC) scores and percentage variances. MDS can project the data into a Euclidean space and preserve the pairwise distance as well as possible. In other words, we finally can treat each root system as a point which has coordinates. The first coordinate is the MDS1. Because the MDS algorithm in MATLAB, cmdscale(), does not randomly map the data, like Principal component analysis (PCA), it will make the MDS1 be the projection which has the most variance. For simplification, we use PC1 instead of MDS1. G_PCs are the coordinates for geodesic distance function. GH_PCs are the coordinates for both geodesic distance and depth functions. To achieve this, we use the new distance matrix which is the square root of the sum of distance matrix with geodesic distance squared and distance matrix with depth squared. Another trait we used is the density estimator of persistence diagram. If we treat the birth value as x, death value as y, then each bar is a 2D point with coordinates (x,y)=(birth, death). Those points including the diagonal line y=x form a persistence diagram. We computed the Gaussian density estimator of those points (not including the line) on the diagram then discretized it into bins (**Supplementary Figure 1C**). More specifically, we first used the Matlab function ksdensity() which is a kernel smoothing function estimate. Given a diagram, the algorithm calculated a default bandwidth for the birth axis, and a default bandwidth for the death axis. The default bandwidth is the optimal for normal densities. The default bandwidths of all the diagrams provide us a bandwidth range. Then we picked an integer (=20) in this range as our fixed bandwidth for the Gaussian KDE in the analysis. We tested if we altered the bandwidth to 10, the correlation between the method with bandwidth=20 and bandwidth=10 for the first 8 PDD_PCs are 0.9996, 0.9986, 0.9893, 0.9673, 0.8770, 0.7715, 0.4091, 0.2214. Choosing very different bandwidths may vary the analysis result. Similarly, we checked the boundary values of all persistence diagrams and determined the overall 2D boundary ([-20, 2000]x[-20,2000]). We picked 20 as the grid resolution which is the same with the bandwidth. We tested whether altering the resolution to 10 would change the first 8 PDD_PCs. We discretized and reshaped the bins into a long vector and performed PCA on these vectors. PDD_PCs are those PCs. In the analysis, we have 301 G_PCs in total and kept 36 G_PCs which occupied 80.05% of variance in the projected Euclidean space. We have 307 GH_PCs and kept 40 GH_PCs which occupied 80.16% of variance. We have 531 PDD_PCs and kept the first 8 PDD_PCs which is 95.45% of the variance. We increased the percentage for PDD_PCs because if we picked 80% of the variance too few PCs are left.

### Correlation analysis, variance inflation factor calculation, PCA and heritability estimation

The R function rcorr() in Hmisc package (Harrell, 2017) was used to compute the significance levels for Pearson correlations analysis. Both the correlation coefficients and the p-value of the correlation for all possible pairs of columns in the data table are returned. To create a graphical display of a correlation matrix and to highlight the most correlated variables (p< 0.05), the function corrplot() in the package of the same name (Wei and Simko, 2017) has been used in the study. Positive correlations are displayed in blue and negative correlations in red color. Color intensity and the size of the circle are proportional to the correlation coefficients (**Supplementary Figure 2A**).

To identify collinearity among explanatory variables, variance inflation factor (VIF) was used. Simply, VIF_j_ = 1/(1-R_j_^2^) where the VIF for variable j is the reciprocal of the inverse of R^2^ from the regression. For each variable, one VIF value is calculated and variables with high values are removed. Instead of picking an arbitrary values in the range of 5-10 that are commonly used, we determined the definition of ‘high’ VIF is the x value at the slope is 1 for fitting a log function (y=a*ln(x-b)+c, where a=15.71, b=0.8853, c=57.88). Thus, the ‘high’ VIF=16.5953. We picked half of the ‘high’ VIF as the ‘median’ VIF (=8) in our study (**Supplementary Figure 2B**). After VIF, 33 univariant traits and 54 PH traits were left for further analysis (**Supplementary Figure 2C**).

The base R function prcomp() was used to perform the PCA with ‘scale =TRUE’ in the study. The number of PCs that captured > 90% variances totally and their rotated data (the scaled data multiplied by the rotation matrix) returned with ‘retx = TRUE’ were retained for subsequent analysis.

Broad sense heritability (**Supplementary Figure 8**) was estimated as follows, H^2^ = Vg/(Vg+Vr/nrep), which is the proportion of genetic variance out of total phenotypic variance. Vg indicates genetic variance, Vr represents the residual variance, while nrep is the mean number of repetitions for each genotype in the experiment. To generate the variance components, the R function lmer() from package lme4 (Bates et al., 2015) was used.

### Genome wide association study and effect size calculation

The multi-locus mixed-model (MLMM) approach was used to perform the genome wide association study. We chose MLMM because root system architecture traits are highly polygenic and MLMM has been demonstrated to identify small effect loci in structured populations that may also have large effect loci, although there is some risk of false-positives (Segura et al., 2012; Baseggio et al., 2021). Both forward and backward stepwise linear mixed-model regressions were used in the model, where the genetic variance and residual variance are estimated before each step. They then are used as follows to obtain generalized least-square (GLS) effect size estimates and F-test P values for each SNP: the SNP with the most significant association is then added to the model as a cofactor for the next step, and the P values for all cofactors are re-estimated together with the variance components. We used both the extended BIC (Bayesian Information Criterion) and the multiple Bonferroni criterion (mBonf) (alpha = 1.0 x e-5) for model selection. The mBonf was used to pick the optimal model. In addition, the maximum model which only includes the forward stepwise regression was also performed with 20 steps to ensure all potential trait-associated SNPs (TAS) were captured. To help determine the colocalization of different TASs, 1Mb window size has been applied which has been commonly used in some other previous studies (Yang et al., 2015b; Hu et al., 2017).

Effect estimates of significant TASs from the optimal model in MLMM were used to calculate the allele effect size. The fractions of the effect estimates and their corresponding average values of the major allele trait were used here to calculate the allele effect size. In this way, the positive value represents the QTL that have increases on the major allele, while negative values indicate QTL that have increases on minor allele.

## Data availability statement

The original contributions presented in the study are included in the article/**Supplementary Material**. All the 3D models can be found at: http://dx.doi.org/10.6084/m9.figshare.23692353. Matlab Code can be found in Li et al., 2019. Raw trait data can be found in **Supplementary Table 5**. Further inquiries can be directed to the corresponding authors.

## Author contributions

ML: Formal analysis, Investigation, Methodology, Software, Visualization, Writing – original draft, Writing – review & editing. ZL: Formal analysis, Investigation, Methodology, Visualization, Writing – original draft, Writing – review & editing. NJ: Formal analysis, Software, Writing – review & editing. BL: Data curation, Writing – review & editing. CT: Resources, Writing – review & editing. SM: Conceptualization, Funding acquisition, Resources, Supervision, Writing – review & editing. CT: Conceptualization, Funding acquisition, Supervision, Writing – original draft, Writing – review & editing.

## Supplementary material

Supplementary Figure 1 Illustrations of persistent homology traits. **(A)** An example of persistence barcode. **(B)** The persistence diagram that is equivalent to the barcode in **(A)**. One example of corresponding bar-to-point is highlighted in pink color. **(C)** Gaussian density estimator of the points on the diagram in **(B)**. Red indicates high density. Blue means low density.

Supplementary Figure 2 Calculations of VIF to remove the redundant traits. **(A)** Pearson’s correlation analysis among all the traits in the study was performed to show the existing redundancies of some measurements. Only significant correlations (p<0.05) were exhibited here. Positive correlations are displayed in blue and negative correlations in red color. Color intensity and the size of the circle are proportional to the correlation coefficients. **(B)** VIF sensitive analysis by fitting log function; **(C)** illustration for VIF with our threshold.

Supplementary Figure 3 Distribution of each VIF remaining trait and first six mPCs.

Supplementary Figure 4 Total lateral root number for IHP1 and ILP1. **(A)** Example images for IHP1 and ILP1 with lateral root highlighted in red window. **(B)** Boxplot of total lateral root number for IHP1 and ILP1. T-test was performed for p value.

Supplementary Figure 5 Venn diagram of profile of trait-associated SNPs (TAS) with no window size across all traits in both MLMM models. **(A)** TASs were identified with optimal model among all different traits. **(B)** More TASs were identified with maximum model among all different traits. Groups of traits were color coded in the figure.

Supplementary Figure 6 Comparison of allele effect size **(A)** and absolute effect size **(B)** among three different classes of traits in the study, multivariate, univariate and PH. Kruskal-Wallis analysis was performed to calculate the p values. The black dot in each violin plot represents the average value of the group of traits.

Supplementary Figure 7 Histogram of number of genotypes for different number of replicates.

Supplementary Figure 8 Broad-sense heritability estimates of all the traits in the study. Groups of traits were color coded.

## References

[B1] Adams H. Emerson T. Kirby M. Neville R. Peterson C. Shipman P.. (2017). Persistence images: A stable vector representation of persistent homology. J. Mach. Learn. Res. 18, 1–35. Available at: https://jmlr.org/papers/v18/16-337.html.

[B2] Amézquita E. J. Quigley M. Y. Ophelders T. Munch E. Chitwood D. H. (2020). The shape of things to come: topological data analysis and biology, from molecules to organisms. Dev. Dyn. 249, 816–833. doi: 10.1002/dvdy.175 32246730 PMC7383827

[B3] Amezquita E. J. Quigley M. Y. Ophelders T. Landis J. B. Koenig D. Munch E and Chitwood D. H. (2021). Measuring hidden phenotype: quantifying the shape of barley seeds using the Euler Characteristic Transform. in silico Plants 4. doi: 10.1093/insilicoplants/diab033

[B4] Atkinson J. A. Pound M. P. Bennett M. J. Wells D. M. (2019). Uncovering the hidden half of plants using new advances in root phenotyping. Curr. Opin. Biotechnol. 55, 1–8. doi: 10.1016/j.copbio.2018.06.002 30031961 PMC6378649

[B5] Baseggio M. Murray M. Wu D. Ziegler G. Kaczmar N. Chamness J.. (2021). Genome-wide association study suggests an independent genetic basis of zinc and cadmium concentrations in fresh sweet corn kernels. G3 11 (8). doi: 10.1093/g3journal/jkab186 PMC8496296 34849806

[B6] Bates D. Mächler M. Bolker B. Walker S. (2015). Fitting linear mixed-effects models using lme4. J. Stat. Software 67, 1–48. doi: 10.18637/jss.v067.i01

[B7] Chazal F. Michel B. (2021). An introduction to topological data analysis: fundamental and practical aspects for data scientists. Front. Artif. Intell. 4. doi: 10.3389/frai.2021.667963 PMC8511823 34661095

[B8] Chen D. Neumann K. Friedel S. Kilian B. Chen M. Altmann T.. (2014). Dissecting the phenotypic components of crop plant growth and drought responses based on high-throughput image analysis. Plant Cell 26, 4636–4655. doi: 10.1105/tpc.114.129601 25501589 PMC4311194

[B9] Cheng J. Sun J. Yao K. Xu M. Cao Y. (2022). A variable selection method based on mutual information and variance inflation factor. Spectrochim Acta A Mol. Biomol Spectrosc 268, 120652. doi: 10.1016/j.saa.2021.120652 34896682

[B10] Chitwood D. H. Topp C. N. (2015). Revealing plant cryptotypes: defining meaningful phenotypes among infinite traits. Curr. Opin. Plant Biol. 24, 54–60. doi: 10.1016/j.pbi.2015.01.009 25658908

[B11] Cignoni P. Calleri M. Corsini M. Dellepiane M. Ganovelli F. Ranzuglia G. (2008). MeshLab: an open-source mesh processing tool. sixth eurographics italian chapter conference, p. 129–136. doi: 10.2312/LocalChapterEvents/ItalChap/ItalianChapConf2008/129-136

[B12] Cohen-Steiner D. Edelsbrunner H. Harer J. (2007). Stability of persistence diagrams. Discrete Comput. Geom. 37, 103–120. doi: 10.1007/s00454-006-1276-5

[B13] Delory B. M. Li M. Topp C. N. Lobet G. (2018). archiDART v3.0: A new data analysis pipeline allowing the topological analysis of plant root systems. F1000Res. 7, 22–10. doi: 10.12688/f1000research.13541.1 29636899 PMC5871803

[B14] De Silva V. Ghrist R. (2007). Homological sensor networks. Notices Am. Math. Soc 54 (1), 10–17. Available at: https://www.ams.org/notices/200701/fea-ghrist.pdf.

[B15] Drew M. C. Saker L. R. Ashley T. W. (1973). Nutrient supply and the growth of the seminal root system in barley: I. THE EFFECT OF NITRATE CONCENTRATION ON THE GROWTH OF AXES AND LATERALS. J. Exp. Bot. 24 (6), 1189–1202. doi: 10.1093/jxb/24.6.1189

[B16] Duc N. T. Ramlal A. Rajendran A. Raju D. Lal S. K. Kumar S.. (2023). Image-based phenotyping of seed architectural traits and prediction of seed weight using machine learning models in soybean. Front. Plant Sci. 14. doi: 10.3389/fpls.2023.1206357 PMC10523016 37771485

[B17] Dudley J. W. Clark D. Rocheford T. R. LeDeaux J. R. (2007). Genetic analysis of corn kernel chemical composition in the random mated 7 generation of the cross of generations 70 of IHP x ILP. Crop Sci. 47, 45–57. doi: 10.2135/cropsci2006.03.0207

[B18] Edelsbrunner H. Letscher D. Zomorodian A. (2002). Topological persistence and simplification. Discrete Comput. Geometry 28 (4), 511–533. doi: 10.1007/s00454-002-2885-2

[B19] Edelsbrunner H. Morozov D. (2013). “Persistent homology: theory and practice,” in Proceedings of the European Congress of Mathematics (Zürich: European Mathematical Society), 31–50.

[B20] Elshire R. J. Glaubitz J. C. Sun Q. Poland J. A. Kawamoto K. Buckler E. S.. (2011). A robust, simple genotyping-by-sequencing (GBS) approach for high diversity species. PloS One 6, e19379. doi: 10.1371/journal.pone.0019379 21573248 PMC3087801

[B21] Falk K. G. Jubery T. Z. O’Rourke J. A. Singh A. Sarkar S. Ganapathysubramanian B.. (2020). Soybean root system architecture trait study through genotypic, phenotypic, and shape-based clusters. Plant Phenomics 2020, 1925495. doi: 10.34133/2020/1925495 33313543 PMC7706349

[B22] Galkovskyi T. Mileyko Y. Bucksch A. Moore B. Symonova O. Price C. A.. (2012). GiA Roots: software for the high throughput analysis of plant root system architecture. BMC Plant Biol. 12, 116. doi: 10.1186/1471-2229-12-116 22834569 PMC3444351

[B23] Gamuyao R. Chin J. H. Pariasca-Tanaka J. Pesaresi P. Catausan S. Dalid C.. (2012). The protein kinase Pstol1 from traditional rice confers tolerance of phosphorus deficiency. Nature 488, 535–539. doi: 10.1038/nature11346 22914168

[B24] Griffiths M. Liu A. E. Gunn S. L. Mutan N. M. Morales E. Y. Topp C. N. (2023). A temporal analysis and response to nitrate availability of 3D root system architecture in diverse pennycress (Thlaspi arvense L.) accessions. Front. Plant Sci. 14. doi: 10.3389/fpls.2023.1145389 PMC10327891 37426970

[B25] Guerra-Urzola R. Van Deun K. Vera J. C. Sijtsma K. (2021). A guide for sparse PCA: model comparison and applications. Psychometrika 86, 893–919. doi: 10.1007/s11336-021-09773-2 34185214 PMC8636462

[B26] Harrell F. R. Jr. (2017). “Hmisc: harrell miscellaneous,” in R package version 4.0-3. Available at: https://CRAN.R-project.org/package=Hmisc.

[B27] Houle D. Govindaraju D. R. Omholt S. (2010). Phenomics: the next challenge. Nat. Rev. Genet. 11 (12), 855–866. doi: 10.1038/nrg2897 21085204

[B28] Hu S. Wang C. Sanchez D. L. Lipka A. E. Liu P. Yin Y.. (2017). Gibberellins promote brassinosteroids action and both increase heterosis for plant height in maize (Zea mays L.). Front. Plant Sci. 8. doi: 10.3389/fpls.2017.01039 PMC5477294 28676808

[B29] Jiang N. Floro E. Bray A. L. Laws B. Duncan K. E. Topp C. N. (2019). Three-dimensional time-lapse analysis reveals multiscale relationships in maize root systems with contrasting architectures. Plant Cell 31, 1708–1722. doi: 10.1105/tpc.19.00015 PMC6713302 31123089

[B30] Kramar M. Goullet A. Kondic L. Mischaikow K. (2013). Persistence of force networks in compressed granular media. Phys. Rev. E Stat. Nonlin Soft Matter Phys. 87 (4), 42207. doi: 10.1103/PhysRevE.87.042207 23679407

[B31] Li P. Chen F. Cai H. Liu J. Pan Q. Liu Z.. (2015). A genetic relationship between nitrogen use efficiency and seedling root traits in maize as revealed by QTL analysis. J. Exp. Bot. 66 (11), 3175–3188. doi: 10.1093/jxb/erv127 25873660 PMC4449538

[B32] Li M. Duncan K. Topp C. N. Chitwood D. H. (2017). Persistent homology and the branching topologies of plants. Am. J. Bot. 104, 349–353. doi: 10.3732/ajb.1700046 28341629

[B33] Li M. Frank M. Coneva V. Mio W. Chitwood D. H. Topp C. N. (2018a). The persistent homology mathematical framework provides enhanced genotype-to-phenotype associations for plant morphology. Plant Physiol. 177, 00104.2018–14. doi: 10.1104/pp.18.00104 PMC6084663 29871979

[B34] Li M. Klein L. L. Duncan K. E. Jiang N. Chitwood D. H. Londo J. P.. (2019). Characterizing 3D inflorescence architecture in grapevine using X-ray imaging and advanced morphometrics: implications for understanding cluster density. J. Exp. Bot. 70, 6261–6276. doi: 10.1093/jxb/erz394 31504758 PMC6859732

[B35] Li Z. Zhang X. Zhao Y. Li Y. Zhang G. Peng Z.. (2018b). Enhancing auxin accumulation in maize root tips improves root growth and dwarfs plant height. Plant Biotechnol. J. 16, 86–99. doi: 10.1111/pbi.12751 28499064 PMC5785362

[B36] Lucas C. J. Zhao H. Schneerman M. Moose S. P. (2013). “Genomic changes in response to 110 cycles of selection for seed protein and oil concentration in maize,” in Seed Genomics (Wiley-Blackwell), 217–236. doi: 10.1002/9781118525524.ch12

[B37] Lynch J. P. (2013). Steep, cheap and deep: an ideotype to optimize water and N acquisition by maize root systems. Ann. Bot. 112 (2), 347–357. doi: 10.1093/aob/mcs293 23328767 PMC3698384

[B38] Medina P. S. Doerge R. W. (2016). TOPOLOGICAL METHODS FOR THE QUANTIFICATION AND ANALYSIS OF COMPLEX PHENOTYPES. Conf. Appl. Stat Agric. doi: 10.4148/2475-7772.1484

[B39] Moose S. P. Dudley J. W. Rocheford T. R. (2004). Maize selection passes the century mark: a unique resource for 21st century genomics. Trends Plant Sci. 9, 358–364. doi: 10.1016/j.tplants.2004.05.005 15231281

[B40] Morse M. (1940). Rank and span in functional topology. Ann. Math. 41, 419–454. doi: 10.2307/1969014

[B41] Pitchers W. Nye J. Márquez E. J. Kowalski A. Dworkin I. Houle D. (2019). A multivariate genome-wide association study of wing shape in drosophila melanogaster. Genetics 211 (4), 1429–1447. doi: 10.1534/genetics.118.301342 30792267 PMC6456314

[B42] Ren W. Zhao L. Liang J. Wang L. Chen L. Li P. (2022). Genome-wide dissection of changes in maize root system architecture during modern breeding. Nat. Plants 8, 1408–1422. doi: 10.1038/s41477-022-01274-z 36396706

[B43] Robinson D. Hodge A. Griffiths B. S. Fitter A. H. (1999). Plant root proliferation in nitrogen–rich patches confers competitive advantage. Proc. R. Soc. London. Ser. B: Biol. Sci. 266 (1418), 431. The Royal Society. doi: 10.1098/rspb.1999.0656

[B44] Schneider H. M. Lor V. S. N. Hanlon M. T. Perkins A. Kaeppler S. M. Borkar A. N.. (2021). Root angle in maize influences nitrogen capture and is regulated by calcineurin B-like protein (CBL)-interacting serine/threonine-protein kinase 15 (ZmCIPK15). Plant Cell Environ. 45, 837–853. doi: 10.1111/pce.14135 PMC9544310 34169548

[B45] Schneider K. L. Xie Z. Wolfgruber T. K. Presting G. G. (2016). Inbreeding drives maize centromere evolution. Proc. Natl. Acad. Sci. United States America 113, E987–E996. doi: 10.1073/pnas.1522008113 PMC4776452 26858403

[B46] Segura V. Vilhjálmsson B. J. Platt A. Korte A. Seren Ü. Long Q.. (2012). An efficient multi-locus mixed-model approach for genome-wide association studies in structured populations. Nat. Genet. 44, 825–830. doi: 10.1038/ng.2314 22706313 PMC3386481

[B47] Stine R. A. (1995). Graphical interpretation of variance inflation factors. Am. Statistician Vol. 49 No. 1 pp, 53–56. doi: 10.2307/2684812

[B48] Symonova O. Topp C. N. Edelsbrunner H. (2015). DynamicRoots: a software platform for the reconstruction and analysis of growing plant roots. PLoS One 10, e0127657. doi: 10.1371/journal.pone.0127657 PMC4452513 26030757

[B49] The MathWorks Inc (2017). MATLAB version: 9.2 (R2017a) (Natick, Massachusetts: The MathWorks Inc).

[B50] Topp C. N. Iyer-Pascuzzi A. S. Anderson J. T. Lee C.-R. Zurek P. R. Symonova O.. (2013). 3D phenotyping and quantitative trait locus mapping identify core regions of the rice genome controlling root architecture. Proc. Natl. Acad. Sci. U. S. A. 110, E1695–E1704. doi: 10.1073/pnas.1304354110 23580618 PMC3645568

[B51] Uga Y. Sugimoto K. Ogawa S. Rane J. Ishitani M. Hara N.. (2013). Control of root system architecture by DEEPER ROOTING 1 increases rice yield under drought conditions. Nat. Genet. 45, 1097–1102. doi: 10.1038/ng.2725 23913002

[B52] Uribelarrea M. Crafts-Brandner S. J. Below F. E. (2009). Physiological N response of field-grown maize hybrids (Zea mays L.) with divergent yield potential and grain protein concentration. Plant Soil 316, 151–160. doi: 10.1007/s11104-008-9767-1

[B53] Uribelarrea M. Moose S. P. Below F. E. (2007). Divergent selection for grain protein affects nitrogen use in maize hybrids. Field Crops Res. 100, 82–90. doi: 10.1016/j.fcr.2006.05.008

[B54] Wei T. Simko V. (2017) R package ‘corrplot’: visualization of a correlation matrix (version 0.84). Available at: https://github.com/taiyun/corrplot.

[B55] White P. J. George T. S. Gregory P. J. Bengough A. G. Hallett P. D. McKenzie B. M. (2013). Matching roots to their environment. Ann. Bot. 112, 207–222. doi: 10.1093/aob/mct123 23821619 PMC3698393

[B56] Yang J. Jiang H. Yeh C.-T. Yu J. Jeddeloh J. A. Nettleton D.. (2015b). Extreme-phenotype genome-wide association study (XP-GWAS): a method for identifying trait-associated variants by sequencing pools of individuals selected from a diversity panel. Plant J. 84, 587–596. doi: 10.1111/tpj.13029 26386250

[B57] Yang C. Lu X. Ma B. Chen S.-Y. Zhang J.-S. (2015a). Ethylene signaling in rice and Arabidopsis: conserved and diverged aspects. Mol. Plant 8, 495–505. doi: 10.1016/j.molp.2015.01.003 25732590

[B58] Ziegler G. Nelson R. Granada S. Krishnan H. B. Gillman J. D. Baxter I. (2018). Genomewide association study of ionomic traits on diverse soybean populations from germplasm collections. Plant Direct 2, e00033. doi: 10.1002/pld3.33 31245681 PMC6508489

[B59] Zou H. Hastie T. Tibshirani R. (2006). Sparse principal component analysis. J. Comput. Graphical Stat 15 (2), 265–286. doi: 10.1198/106186006X113430

