# Supplementary material for: Topological data analysis expands the genotype to phenotype map for 3D maize root system architecture
Source: Front Plant Sci. 2024 Jan 15;14:1260005. doi: 10.3389/fpls.2023.1260005 (PMC10822944; doi:10.3389/fpls.2023.1260005)
Supplement: Supplementary Figure 1 — Illustrations of persistent homology traits. (A) An example of persistence barcode. (B) The persistence diagram that is equivalent to the barcode in (A). One example of corresponding bar-to-point is highlighted in pink color. (C) Gaussian density estimator of the points on the diagram in (B). Red indicates high density. Blue means low density. [file DataSheet_1.zip › Supplementary Figure 5.PDF]

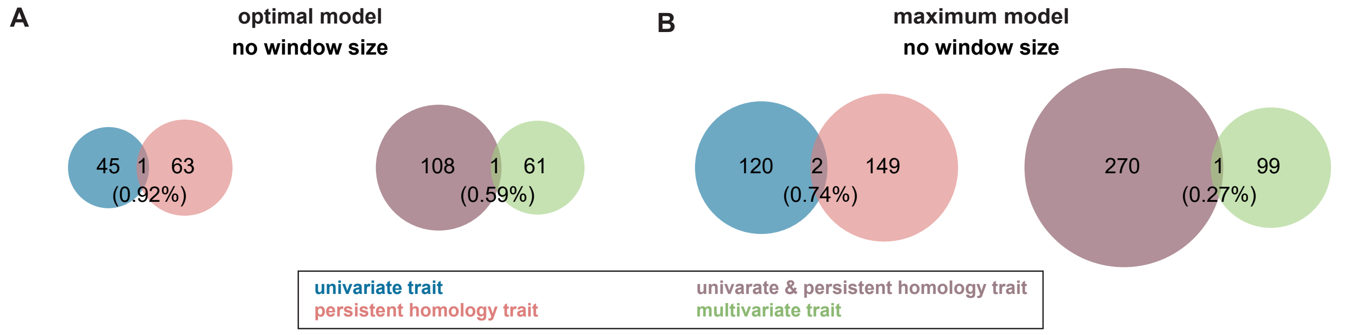
A
optimal model
no window size
45
1
63
(0.92%)
108
1
61
(0.59%)
B
maximum model
no window size
120
2
149
(0.74%)
270
1
99
(0.27%)
univariate trait
persistent homology trait
univarate & persistent homology trait
multivariate trait
