# Supplementary figures and images for: Topological data analysis expands the genotype to phenotype map for 3D maize root system architecture
Source: Front Plant Sci. 2024 Jan 15;14:1260005. doi: 10.3389/fpls.2023.1260005 (PMC10822944; doi:10.3389/fpls.2023.1260005)

**A** persistence barcode

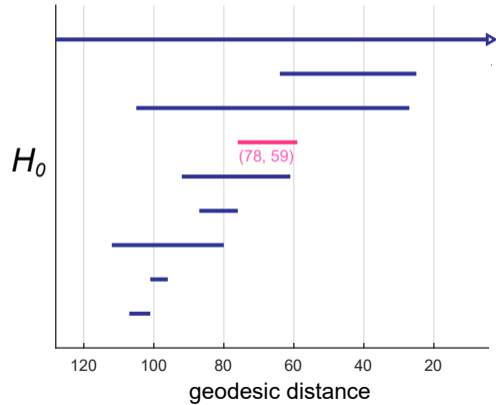

**B** persistence diagram

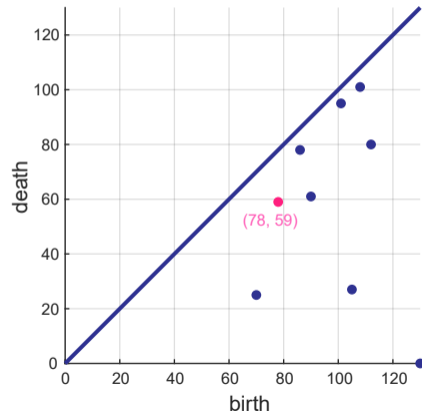

**C** Gaussian density estimator

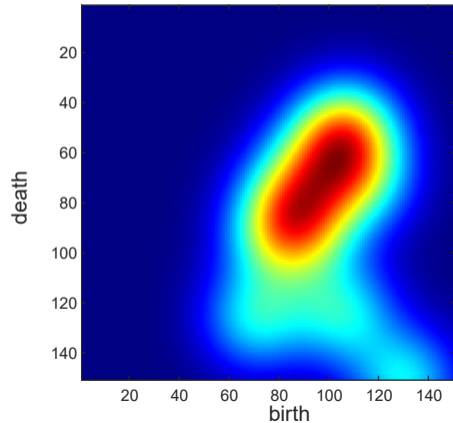

Supplement: Supplementary Figure 1 — Illustrations of persistent homology traits. (A) An example of persistence barcode. (B) The persistence diagram that is equivalent to the barcode in (A). One example of corresponding bar-to-point is highlighted in pink color. (C) Gaussian density estimator of the points on the diagram in (B). Red indicates high density. Blue means low density. [file DataSheet_1.zip › Supplementary Figure 1.PDF]

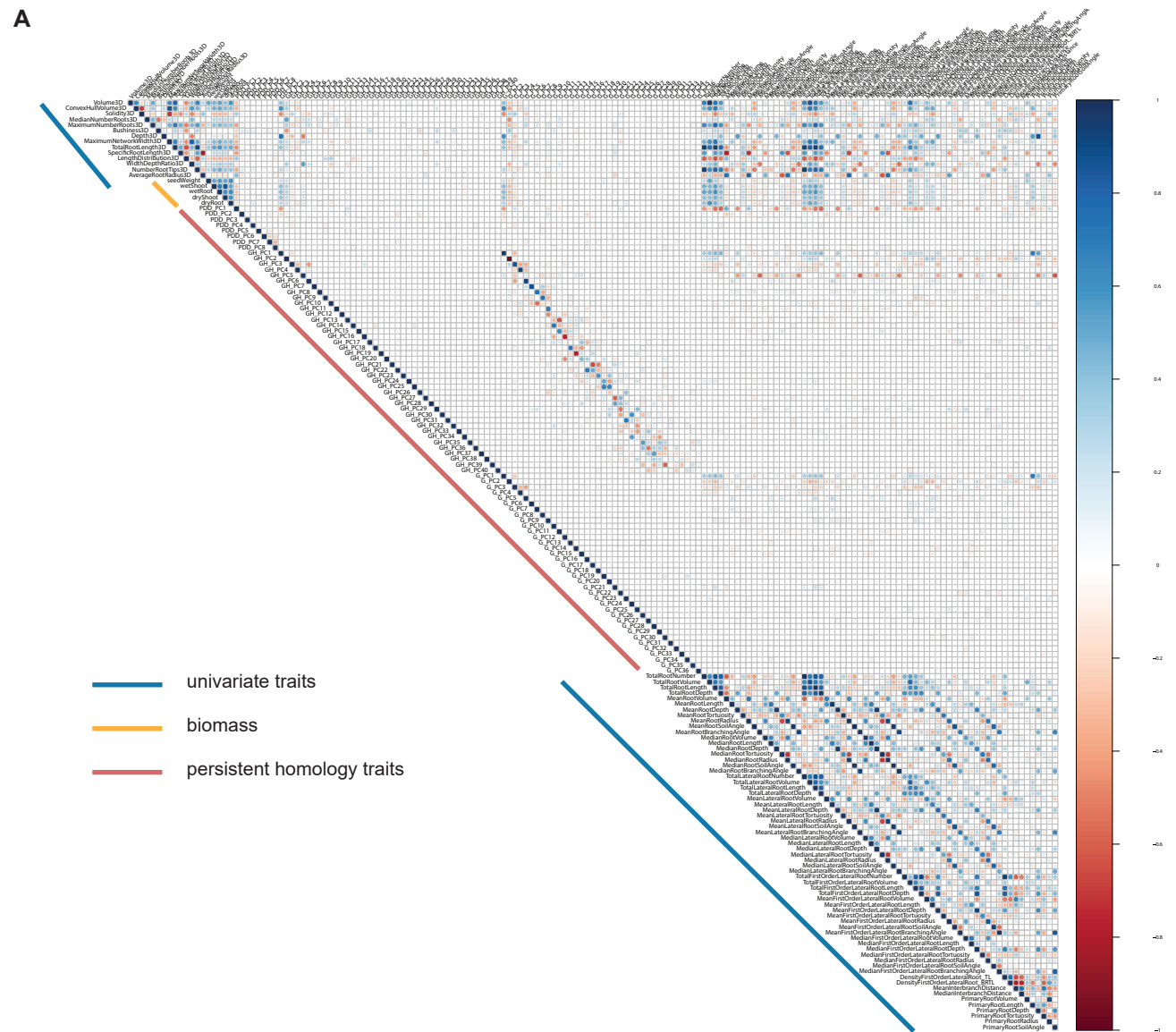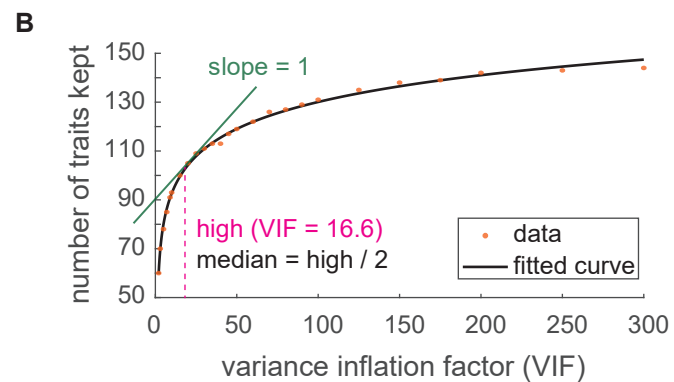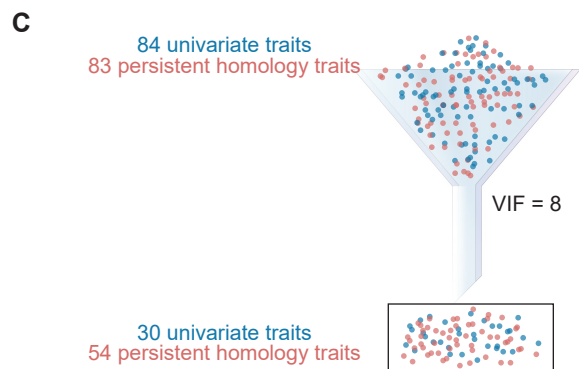

Supplement: Supplementary Figure 1 — Illustrations of persistent homology traits. (A) An example of persistence barcode. (B) The persistence diagram that is equivalent to the barcode in (A). One example of corresponding bar-to-point is highlighted in pink color. (C) Gaussian density estimator of the points on the diagram in (B). Red indicates high density. Blue means low density. [file DataSheet_1.zip › Supplementary Figure 2.PDF]

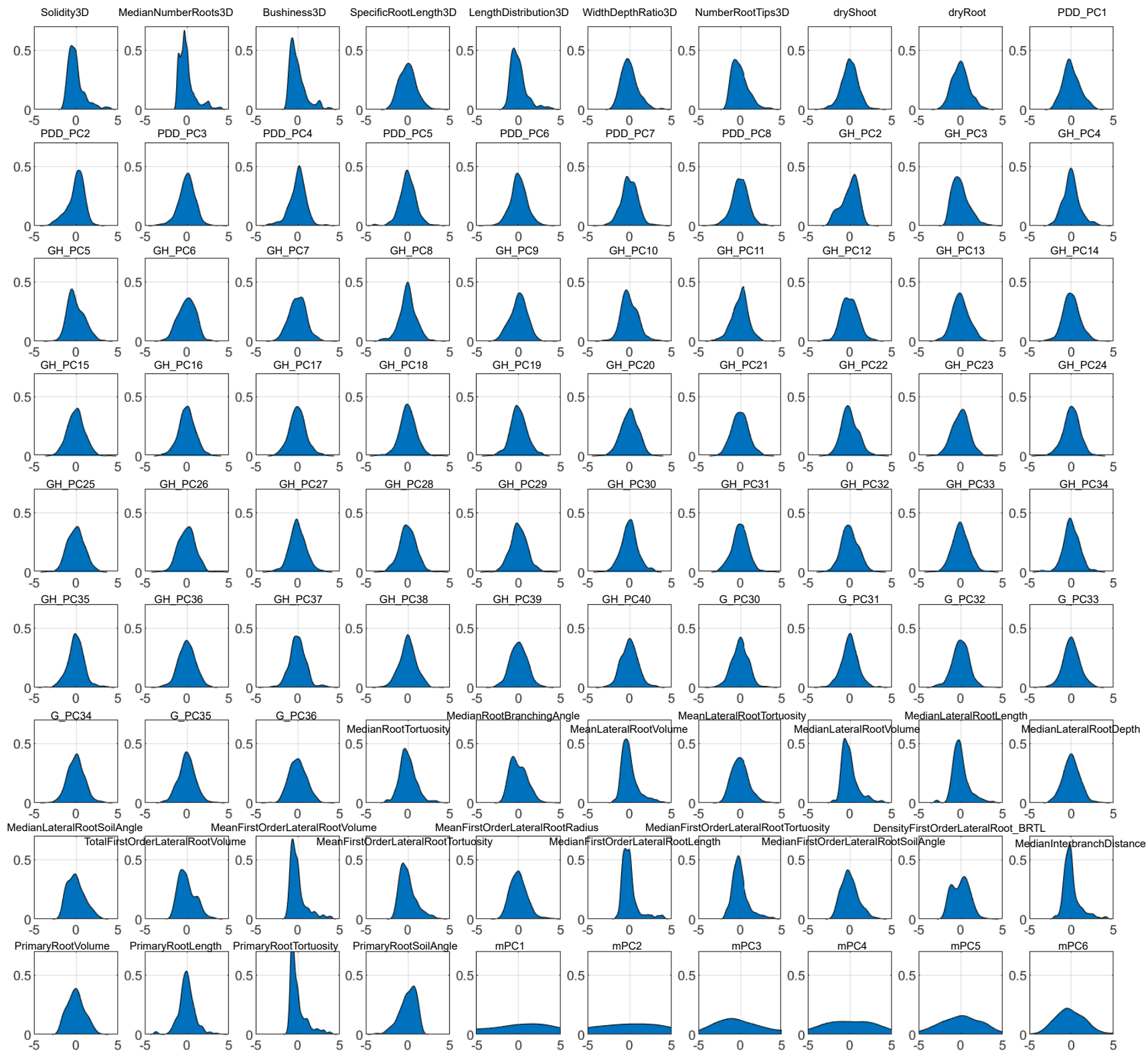

Supplement: Supplementary Figure 1 — Illustrations of persistent homology traits. (A) An example of persistence barcode. (B) The persistence diagram that is equivalent to the barcode in (A). One example of corresponding bar-to-point is highlighted in pink color. (C) Gaussian density estimator of the points on the diagram in (B). Red indicates high density. Blue means low density. [file DataSheet_1.zip › Supplementary Figure 3.PDF]

**A**

Illinois High Protein    Illinois Low Protein

(IHP1)

(ILP1)

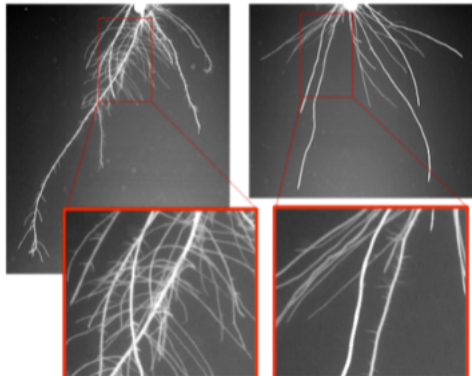**B**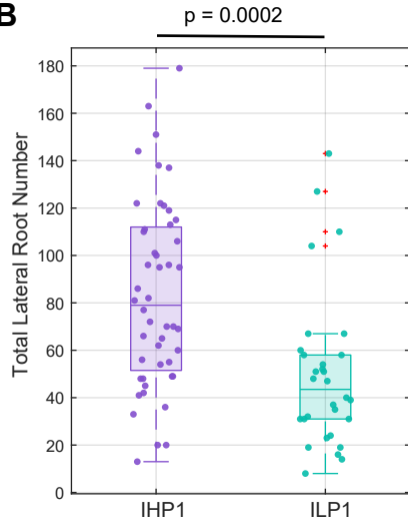

Supplement: Supplementary Figure 1 — Illustrations of persistent homology traits. (A) An example of persistence barcode. (B) The persistence diagram that is equivalent to the barcode in (A). One example of corresponding bar-to-point is highlighted in pink color. (C) Gaussian density estimator of the points on the diagram in (B). Red indicates high density. Blue means low density. [file DataSheet_1.zip › Supplementary Figure 4.PDF]

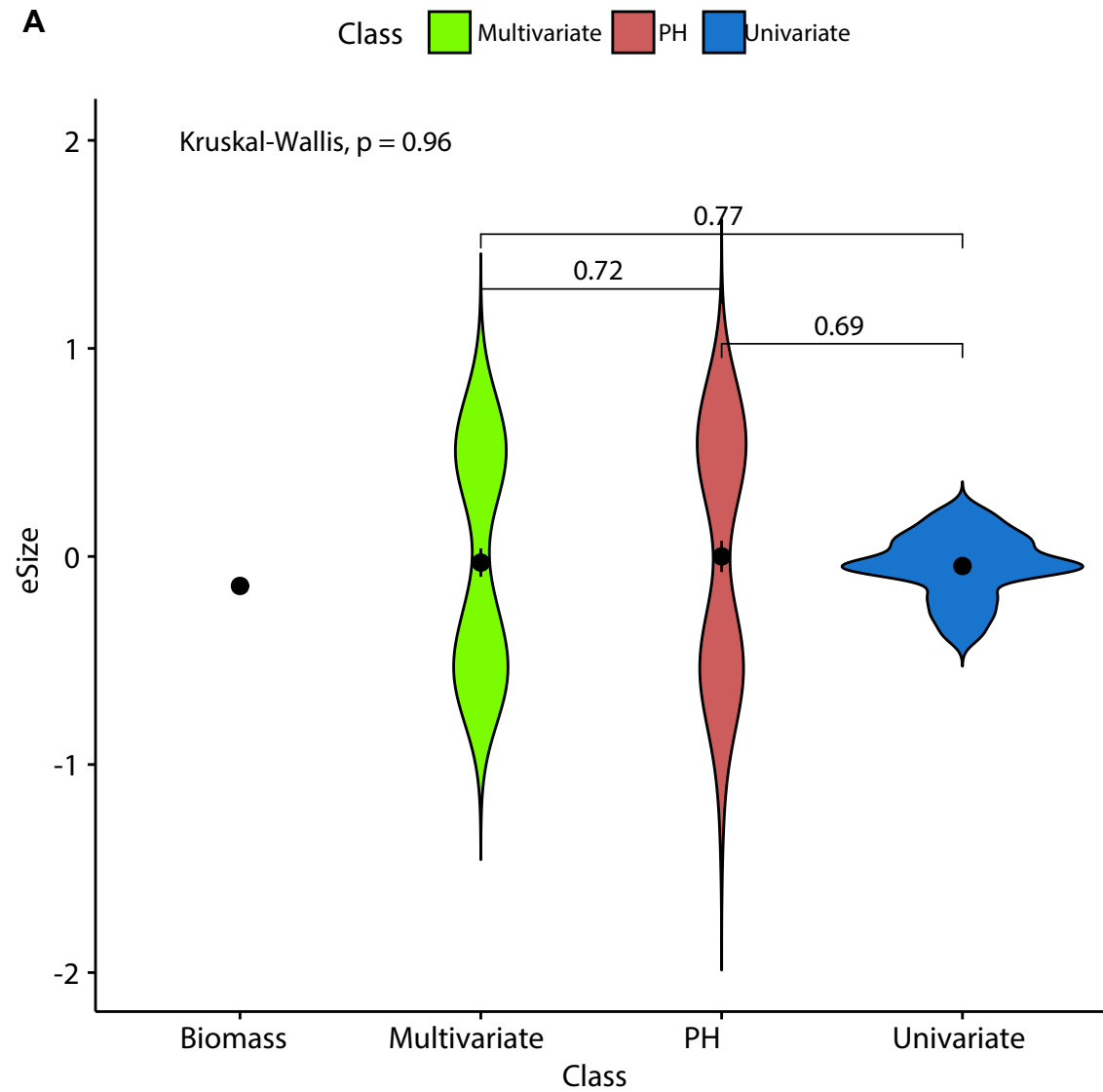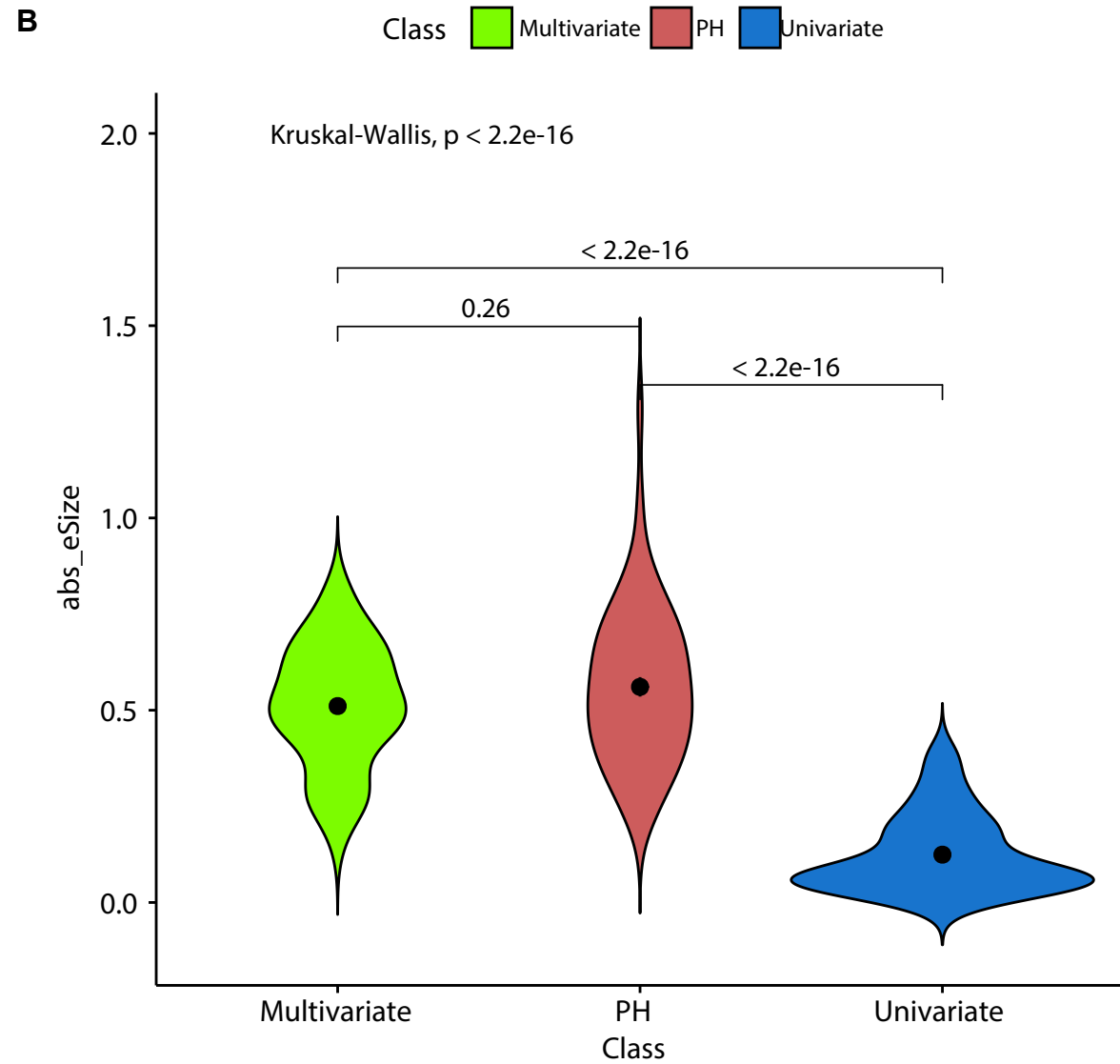

Supplement: Supplementary Figure 1 — Illustrations of persistent homology traits. (A) An example of persistence barcode. (B) The persistence diagram that is equivalent to the barcode in (A). One example of corresponding bar-to-point is highlighted in pink color. (C) Gaussian density estimator of the points on the diagram in (B). Red indicates high density. Blue means low density. [file DataSheet_1.zip › Supplementary Figure 6.PDF]

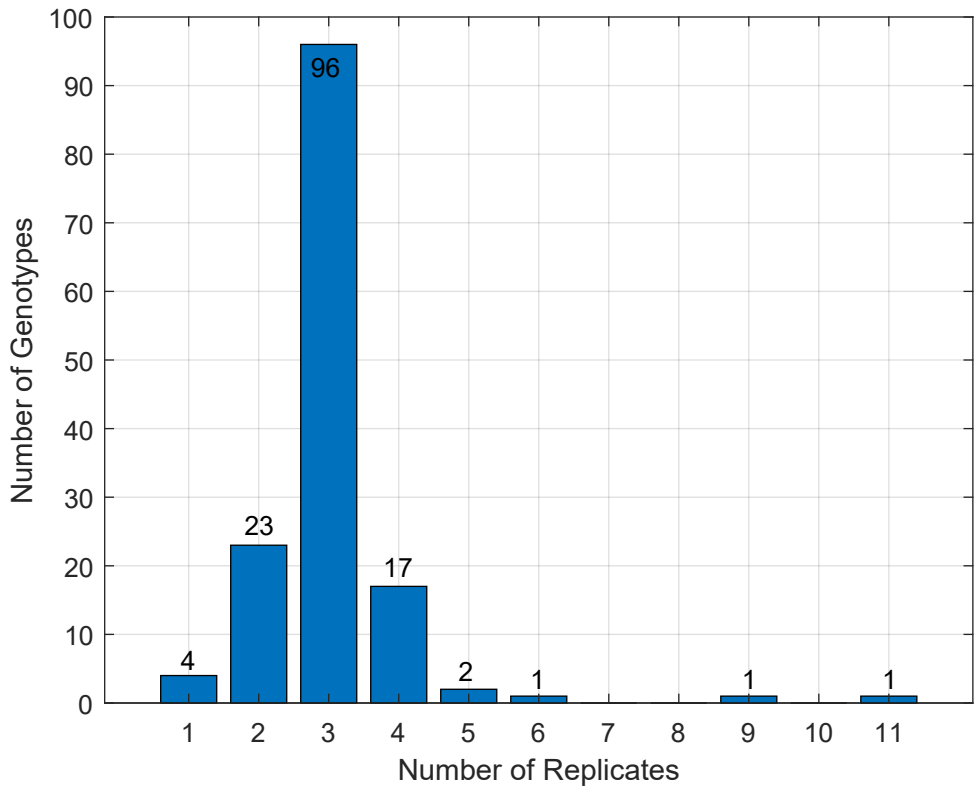

Supplement: Supplementary Figure 1 — Illustrations of persistent homology traits. (A) An example of persistence barcode. (B) The persistence diagram that is equivalent to the barcode in (A). One example of corresponding bar-to-point is highlighted in pink color. (C) Gaussian density estimator of the points on the diagram in (B). Red indicates high density. Blue means low density. [file DataSheet_1.zip › Supplementary Figure 7.PDF]

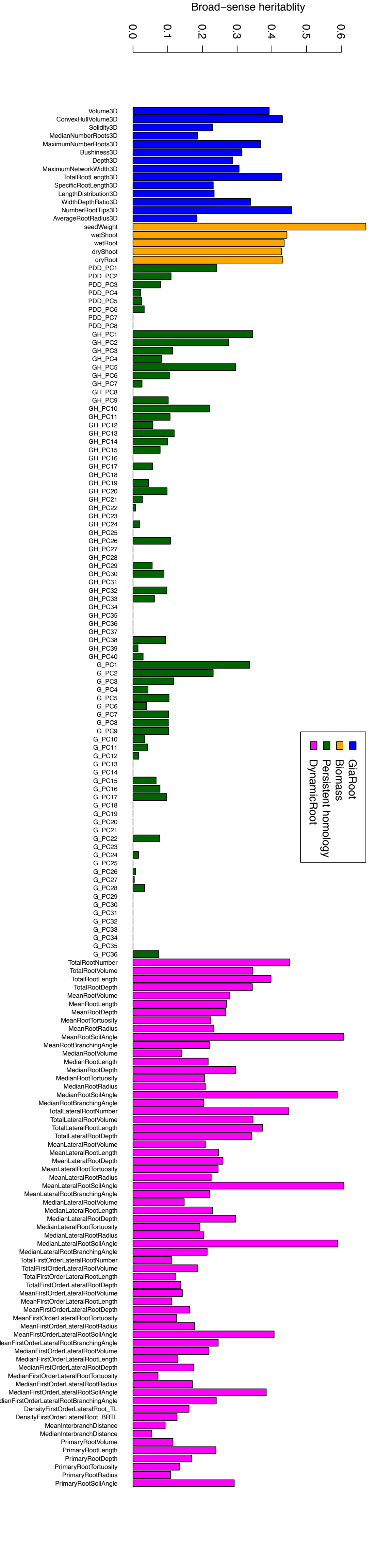

Supplement: Supplementary Figure 1 — Illustrations of persistent homology traits. (A) An example of persistence barcode. (B) The persistence diagram that is equivalent to the barcode in (A). One example of corresponding bar-to-point is highlighted in pink color. (C) Gaussian density estimator of the points on the diagram in (B). Red indicates high density. Blue means low density. [file DataSheet_1.zip › Supplementary Figure 8.PDF]
